# Does Drying Affect Gel Networks?

**DOI:** 10.3390/gels4020032

**Published:** 2018-04-03

**Authors:** Dave J. Adams

**Affiliations:** School of Chemistry, University of Glasgow, Glasgow G12 8QQ, UK; dave.adams@glasgow.ac.uk

**Keywords:** gels, drying, microscopy, scattering

## Abstract

The properties of low molecular weight gels are determined by the underlying, self-assembled network. To access information on the network, it is common for techniques to be used that require the gel to be dried, such as transmission electron microscopy or scanning electron microscopy. The implicit assumption is that this drying has no bearing on the data collected. Here, we discuss the validity of this assumption.

## 1. Introduction

Low molecular weight gels (LMWG) consist mainly of a solvent [1,2]. The solvent is immobilized by a matrix, which is formed by the low molecular weight gelator molecules self-assembling into a three-dimensional matrix. Such gels contain certainly less than 5% *w*/*v* of the gelator, perhaps typically less than 1% *w*/*v* and have been reported at even lower concentrations. However, it is the network that gives the gel its properties such as stability to inversion, rigidity, and perhaps thixotropy. To understand the gels therefore, it is necessary to understand the network. In this short paper, we aim to highlight potential opportunities and issues in this area. By design, we have not attempted to reference widely, but hope that this will be thought-provoking.

There are many examples where information has been gathered at the molecular length scale, for example using infra-red or nuclear magnetic resonance spectroscopy to access information about interactions leading to the self-assembly such as hydrogen bonding [3,4]. Whilst useful, these techniques usually probe the molecule–molecule interactions, but do not provide direct information about the network. 

It is common to utilize two broad approaches to understand the networks. First, the mechanical properties of the gels can be measured in some way. Methods such as rheology, microrheology, and melting points can be used to compare and understand the gels [4,5]. For example, absolute values of the storage modulus (G’) and loss modulus (G”) allow comparison between gels, and there are a number of reports where the relationship between G’ and the gelator concentration can be used to infer some aspect of the underlying network [6,7]. 

A second approach is to examine the structures that form the networks. Most commonly, these structures are long, one-dimensional structures, and there are reports of fibers, tubes, helical fibers, tapes, and bundles being formed. The networks are formed by these structures that must entangle, branch or otherwise form cross-links to provide the network. The properties of the gels will be determined by parameters such as the type of assembled structure, the mechanical properties of these structures (such as modulus, persistence length etc.), the propensity for bundling and lateral association, the type of cross-links and the number of the cross-links. Hence, to understand the properties, we would ideally be able to determine all of these. 

At this point, many different techniques are used. These include a range of microscopy techniques including transmission electron microscopy (TEM), scanning electron microscopy (SEM), atomic force microscopy (AFM), and confocal microscopy [3,4]. A number of scattering tools have also been used such as small angle X-ray scattering (SAXS) or small angle neutron scattering (SANS) [8]. A key and important question here arises from the techniques used and the underlying assumption that is often made: does drying affect the network?

## 2. How Does Drying Affect the Networks

This question arises because many of the techniques described require that the gels are dried in some way. For example, TEM and SEM are usually carried out under high vacuum, and AFM most often is carried out on a dried sample on a surface. Further complications can arise; for example, it is common to add a heavy metal salt as a stain to aid the imaging of the structures in TEM due to the low contrast of the gelators, which tend not to be electron rich. Additionally, in some cases where mixed solvent systems are used, drying does not necessarily result in both solvents being removed at the same rate, which may lead to structural changes. Despite these potential issues, it is rare for these to be acknowledged. Rather, data are provided with the implicit assumption that they represent the gel in the solvated state. The potential for artefacts is stated in reviews [9] but rarely acknowledged in the literature. A recent review on aspects of microscopy provides some example drying artefacts [10], which can also be seen in many gel papers. We have deliberately not provided examples here as it would be unfair to single out specific instances when these are widespread in the field.

On top of this, there is also the issue that any microscopy technique will only capture a tiny fraction of the sample. It has been calculated for example that it would take a billion years to study the volume of material contained within a little finger by TEM [11]. By default, this means one is likely to miss important details, or know whether the image presented is representative of the sample for example.

**Figure 1:**
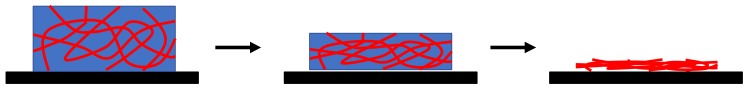
Simplified cartoon showing the collapse of a 3D network to a 2D film on drying.

It is not clear here why this should be the case; the networks represent by far a smaller fraction of the gel compared to the solvent. Hence, at the least, drying will result in the collapse of the gel and then the best-case scenario will be an essentially two-dimensional representation of what used to be a three-dimensional network (Figure 1). Furthermore, the network is formed by the self-assembly of the gelator. This is driven essentially by the gelator no longer being dissolved in the solvent; as such, gelator–gelator interactions dominate over the gelator–solvent interactions. As the concentration increases, lateral association and bundling are more likely to occur. Drying leads to an increase in concentration and so, coupled with the gelator–gelator interactions, bundling will be expected. There is increasing realization that the interactions between the substrate and gel network can be important. This can be shown in the gel state and also by differences in the structures imaged by different techniques where the gels have been dried down on to different surfaces. 

As a side note here, it also seems surprising that there are many examples where powder X-ray diffraction data are collected on dried gels and the data used to infer information about packing in the gel state. Again, concentration and drying effects, as well as surface interactions, may occur. There are examples where it has categorically been demonstrated that the packing in the gel state and that in crystals grown from the gel state are not the same. Again, the assumption that the data for dried gels represents that, for the wet gels, is often allowed to pass without question. 

Because of all of the above, it seems a fair requirement that, rather than simply providing images where gels have been dried with no comment, at the very least there should be included evidence that there have been no changes on drying. This may seem dramatic, but it is usually not clear why the assumption that drying has not led to artefacts is valid. My personal experience is also that referees will usually request that these data are added if they are not included in the original manuscript, with little evidence that this will add valid information. 

So, following this, the obvious next question is how might one show that the data for dried gels are valid? There are examples where different drying techniques have been used in an effort at minimizing drying effects. Here, at the very least, if there are differences between the data collected on gels dried in different ways, it seems impossible not to acknowledge that there can be drying effects! Certainly, the standard inclusion of data collected using different imaging techniques would be a significant improvement to the field. A question might arise as to how close the images have to be to consider these to represent the real structures. This is of course difficult as there are many types of gels and many techniques, but a first question could be “do any of the images represent the real structures”, followed by an analysis as to how the structures apparently differ. Surface interactions and chemistry are known to be able to affect gel structures [12], so drying on different surfaces could provide alternative structures, of course none of which represent that in the gel phase. An analysis of the sizes of structures can be useful here; if the gels are translucent, it is highly unlikely that these consist of structures that are microns in size in each dimension for example. As a final note, including images at a range of magnifications to demonstrate homogeneity is also the best practice, along with addition of images in the Supporting Information to show that those that are “representative” are indeed truly so.

Additionally, data can be collected on wet gels. For example, we have tended to use confocal microscopy for this reason as images can be collected in the gel state. There are also drawbacks here as well of course (most commonly that a stain needs to be added), but the advantage of being able to image the network wet seems important. Perhaps a greater drawback is the magnification that can be achieved, although super-resolution techniques are becoming more common. Cryo-TEM can be used to image the gels in the solvated state. Again, there are drawbacks in that thin films are used, so it is often only possible to infer information about the primary structures as opposed to the gel networks. Artefacts are again also possible [10]. Cryo-SEM has also been used for some gels. Artefacts are again possible as the sample has to be frozen before analysis [13].

Scattering techniques such as SAXS and SANS have strong advantages here. They can be used on the gels themselves and the data can be used to access information about the primary structures and the networks depending on the length scale of the experiment. There are of course also drawbacks. Typically, access to a large-scale facility is needed, although lab-scale X-ray sources are becoming more common and effective. Furthermore, the data typically have to be fitted to a model to infer information. However, the scattering data can also be collected on bulk gels, allowing one to access information on millions of structures, as opposed to TEM or SEM where perhaps a hundred structures at most are imaged. Despite this, it is common in our experience for referees to ask for microscopy even if scattering is utilized to “prove” the structures. 

**Figure 2:**
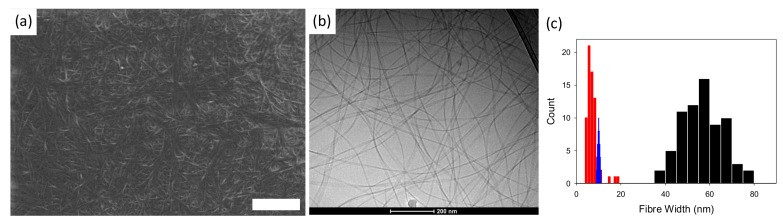
(**a**) SEM image of an air-dried xerogel of a dipeptide-based low molecular weight gel; (**b**) Cryo-TEM of the gel prior to drying; (**c**) Histogram of the widths of fibers measured from the SEM images (black) and cryo-TEM (red), along with the distribution expected from a Gaussian distribution (generated from SigmaPlot with a standard deviation of 0.4) around the mean diameter determined by SANS (blue). The data are re-drawn from those shown in reference [14].

As a single specific example to exemplify the issues discussed here, we recently showed that the data from SANS and cryo-TEM were in close agreement in terms of fiber widths in a low molecular weight, functionalized dipeptide-based hydrogel (Figure 2) [14]. We were able to follow the drying process using contrast-matching SANS experiments and showed that the structures were similar when dried. However, on imaging a dried gel by SEM, the fiber widths were significantly greater than in the wet (as imaged by cryo-TEM) or wet and dry by SANS. Our interpretation was that the fibers bundled on drying leading to the far greater diameters imaged by SEM, but that there was sufficient contrast that the SANS experiments were able to probe the primary structures. From experience with such materials, it is very common for the SEM images to show structures that are far larger than expected from SAXS or SANS data. As mentioned above, this is not surprising; the structures are hydrophobic and so concentration would be expected to lead to the hydrophobic structures coming closer together and hence likely to associate. 

## 3. Conclusions

We are of course not suggesting that there are artefacts in all images of gels, nor that all such data should be discounted. Rather, we would like to suggest to the community as a whole that the question be asked as to whether there is any evidence that there are no drying effects when collecting data. Is there any supporting data that shows that the structures imaged are as in the wet gel? If not, perhaps we should move to a situation where such data is therefore not included or, if it is to be added, serious caveats are acknowledged.
